# Supplementary material for: The metabolic hormone adiponectin affects the correlation between nutritional status and pneumococcal vaccine response in vulnerable indigenous children
Source: PLoS One. 2022 Jul 21;17(7):e0270736. doi: 10.1371/journal.pone.0270736 (PMC9302759; doi:10.1371/journal.pone.0270736)
Supplement: S1 Table — (DOCX) [file pone.0270736.s001.docx]

**S1 Table: Number and percentage of children with a serotype specific post vaccination IgG (µg/ml) response below 0.35 µg/ml, stratified by nutritional status.**

|  | **Total (210)** | | **Stunting (80)** | | **Normal weight (81)** | | **Overweight (49)** | |  |
| --- | --- | --- | --- | --- | --- | --- | --- | --- | --- |
| **Serotype** | **N <0.35 µg/ml** | **% <0.35 µg/ml** | **N <0.35 µg/ml** | **% <0.35 µg/ml** | **N <0.35 µg/ml** | **% <0.35 µg/ml** | **N <0.35 µg/ml** | **% <0.35 µg/ml** | **p-value** |
| 1 | 29 | 13,8% | 11 | 13,8 % | 12 | 14,8% | 6 | 12,2% | 0.97 |
| 3 | 4 | 1,9% | 2 | 2,5% | 1 | 1,2% | 1 | 2% | 0.84 |
| 4 | 42 | 20% | 17 | 21,3% | 14 | 17,3% | 11 | 22,4% | 0.74 |
| 5 | 26 | 12,4% | 12 | 15% | 9 | 11,1% | 5 | 10,2% | 0.72 |
| 6a | 37 | 17,6% | 13 | 16,3% | 14 | 17,3% | 10 | 20,4% | 0.87 |
| 6b | 99 | 47,1% | 40 | 50% | 38 | 46,9% | 21 | 42,9% | 0.73 |
| 7f | 1 | 0,5% | 1 | 1,3% | 0 | 0% | 0 | 0% | 0.61 |
| 9v | 43 | 20,5% | 14 | 17,5% | 17 | 21% | 12 | 24,5% | 0.63 |
| 14 | 22 | 10,5% | 9 | 11,3% | 5 | 6,2% | 8 | 16,% | 0.18 |
| 18c | 18 | 8,6% | 7 | 8,8% | 5 | 6,2% | 6 | 12,2% | 0.47 |
| 19a | 17 | 8,1% | 9 | 11,3% | 4 | 4,9% | 4 | 8,2% | 0.32 |
| 19f | 9 | 4,3% | 4 | 5% | 3 | 3,7% | 2 | 4,1% | 0.91 |
| 23f | 55 | 26,2% | 25 | 31,3% | 18 | 22,2% | 12 | 24,5% | 0.42 |
| Mean IgG | 5 | 2,4% | 3 | 3,8% | 1 | 1,2% | 1 | 2% | 0.74 |

A p-value <0.05 is considered statistically significant.
