# Supplementary material for: The metabolic hormone adiponectin affects the correlation between nutritional status and pneumococcal vaccine response in vulnerable indigenous children
Source: PLoS One. 2022 Jul 21;17(7):e0270736. doi: 10.1371/journal.pone.0270736 (PMC9302759; doi:10.1371/journal.pone.0270736)
Supplement: Attachment — Submitted filename: Response to Reviewers.docx [file pone.0270736.s002.docx]

Dear dr. Magni,

We thank you for your constructive comments and suggestions. Your comments are highly appreciated, and we have incorporated the provided feedback accordingly. The revised manuscript has been uploaded; please find our response to each specific comment below.

*Journal requirements*

1. Please ensure that your manuscript meets PLOS ONE's style requirements, including those for file naming. The PLOS ONE style templates can be found at https://journals.plos.org/plosone/s/file?id=wjVg/PLOSOne_formatting_sample_main_body.pdf and https://journals.plos.org/plosone/s/file?id=ba62/PLOSOne_formatting_sample_title_authors_affilaffilaf.pdf

We have adjusted the manuscript style according to the PLOS ONE’s style requirements.

2. Please provide the catalog numbers and sources of all kits referred to in lines 177-183.

We have added the catalog numbers and sources of all kits to the ‘sampling and laboratory methods’ section.

3. Please provide the registration information for the original clinical study.

The original study was registered in a primary registry of the World Health Organization (ICTRP / RPCEC) with identifier number RPCEC00000158. We have included this in the methods section.

4. Please list the names of the nine indigenous Warao communities where participants were from.

The nine indigenous Warao communities are named in the revised version of our manuscript.

5. Thank you for stating the following in the Financial Disclosure section:

[The original study was supported by Pfizer Venezuela and the Fundacion para la Investigación en Micobacterias, Caracas, Venezuela. Lilly M. Verhagen was supported by a Clinical Research Talent fellowship of the UMC Utrecht, The Netherlands. The funders had no role in study design, data collection and analysis, decision to publish, or preparation of the manuscript.].

We note that one or more of the authors have an affiliation to the commercial funders of this research study: Pfizer Venezuela

1. Please provide an amended Funding Statement declaring this commercial affiliation, as well as a statement regarding the Role of Funders in your study. If the funding organization did not play a role in the study design, data collection and analysis, decision to publish, or preparation of the manuscript and only provided financial support in the form of authors' salaries and/or research materials, please review your statements relating to the author contributions, and ensure you have specifically and accurately indicated the role(s) that these authors had in your study. You can update author roles in the Author Contributions section of the online submission form.

Please also include the following statement within your amended Funding Statement.

“The funder provided support in the form of salaries for authors [insert relevant initials], but did not have any additional role in the study design, data collection and analysis, decision to publish, or preparation of the manuscript. The specific roles of these authors are articulated in the ‘author contributions’ section.”

If your commercial affiliation did play a role in your study, please state and explain this role within your updated Funding Statement.

None of the authors has or had an affiliation to Pfizer Venezuela. Jacobus de Waard has received funding from Pfizer Venezuela for the here presented study. However, the funder did not provide support in the form of salaries for authors, nor did they have any additional role in the study design, data collection and analysis, decision to publish, or preparation of the manuscript.

Pfizer Venezuela only provided financial support in the form of vaccine (PCV13) supply and research materials.

The specific roles of the authors are articulated in the ‘author contributions’ section.

2. Please also provide an updated Competing Interests Statement declaring this commercial affiliation along with any other relevant declarations relating to employment, consultancy, patents, products in development, or marketed products, etc.

Within your Competing Interests Statement, please confirm that this commercial affiliation does not alter your adherence to all PLOS ONE policies on sharing data and materials by including the following statement: "This does not alter our adherence to PLOS ONE policies on sharing data and materials.” (as detailed online in our guide for authors http://journals.plos.org/plosone/s/competing-interests). If this adherence statement is not accurate and there are restrictions on sharing of data and/or materials, please state these. Please note that we cannot proceed with consideration of your article until this information has been declared.

There were no competing interests, as clarified above. We have also included this in the updated Funding Statement and Competing Interest Statement in our cover letter.

6. We note that you have indicated that data from this study are available upon request. PLOS only allows data to be available upon request if there are legal or ethical restrictions on sharing data publicly. For information on unacceptable data access restrictions, please see http://journals.plos.org/plosone/s/data-availability#loc-unacceptable-data-access-restrictions.

In your revised cover letter, please address the following prompts:

a) If there are ethical or legal restrictions on sharing a de-identified data set, please explain them in detail (e.g., data contain potentially identifying or sensitive patient information) and who has imposed them (e.g., an ethics committee). Please also provide contact information for a data access committee, ethics committee, or other institutional body to which data requests may be sent.

b) If there are no restrictions, please upload the minimal anonymized data set necessary to replicate your study findings as either Supporting Information files or to a stable, public repository and provide us with the relevant URLs, DOIs, or accession numbers. Please see http://www.bmj.com/content/340/bmj.c181.long for guidelines on how to de-identify and prepare clinical data for publication. For a list of acceptable repositories, please see http://journals.plos.org/plosone/s/data-availability#loc-recommended-repositories.

We will update your Data Availability statement on your behalf to reflect the information you provide.

We have uploaded the data in accordance with the provided guidelines and adjusted the text accordingly. The data are now freely available via the following URL: <https://datadryad.org/stash/share/LUfQ9WNDrEOw5Aq9YIpEcugbAU7FHfyE0oVmK_abeC4>

However, please note that this is a temporary URL because the status of our project is currently set to ‘private for peer review’. We will replace this URL with a definite one upon acceptance of our manuscript.

7. Please include captions for your Supporting Information files at the end of your manuscript, and update any in-text citations to match accordingly. Please see our Supporting Information guidelines for more information: http://journals.plos.org/plosone/s/supporting-information.

We have added the captions for our supporting table at the end of our manuscript.

*Reviewers’ comments*

1. Figures 2A-2C only showed the simple regression lines of IgG levels ~ metabolic hormone levels. The data points should also be plotted with different color /shape for the three categories. The significance levels of the interaction terms in the multiple regression models should be reported here to quantitatively show whether the correlations are different between categories.

We have adjusted the figures 2A-2C. The figures now also include the plotted data points in addition to the regression lines. The significance levels of the interaction terms in the multiple regression models are added to the legend of the figures.

2. Table 1, it’s still unclear to me how to interpret the different superscript letters. Please be more specific.

Significantly different median values in the same row are indicated with different superscript letters, while median values that are not significantly different are indicated with identical superscript letters. We have further clarified the interpretation of the superscript letters in the footnote.

3. Table 2, it is unclear the GMRs were between which two groups. For example, for All children category, how was GMR calculated? If it’s a ratio, what are numerator and denominator, respectively? Note that for results in Table 3, it’s clear the comparison is between under-/over-nutrition group and normal group. However, it’s unclear here.

We indeed noted that our presentation of the results was unclear using a GMR. Therefore, we have now used linear regression coefficients for the presentation of the results in tables 2 and 3.

We have also clarified the comparator groups in the methods section. To avoid over-adjusting, we did not include all malnutrition measures in the same regression model, but instead created two different regression models for undernutrition and overnutrition. We clarified how we analysed malnutrition indicators in the Methods section of the revised manuscript.

4. Table 3, note that unadjusted GMR are the same for under- and over nutrition groups, and it’s the same as that in Table 2 (1.11, 1.06, and 1.01). Again, please clarify.

We thank the reviewer for pointing this out. The unadjusted GMR were the same for all variables, except for nutritional status, because these represented the univariate correlations between e.g. metabolic hormones and antibody concentrations. To be comprehensive, we had initially added these univariate analysis to both tables. However, we acknowledge the unclarity this may have caused and we have now removed the GMR and added linear regression coefficient, in response to the previous comment.

Hence, we have changed tables 2 and 3 so that the stratified univariate analysis is shown in table 2 while table 3 now contains the multivariable linear GEE models.

5. In the main text, GMR was interpreted as OR, which is incorrect.

We thank the reviewer for this observation. Since we now use linear regression coefficients in response to the comments above, we adjusted the text accordingly in the results section.

We hope that our modifications render our revised manuscript suitable for publication in PLOS ONE.

Sincerely,

On behalf of all authors,

Lilly M. Verhagen
