# Supplementary material for: The metabolic hormone adiponectin affects the correlation between nutritional status and pneumococcal vaccine response in vulnerable indigenous children
Source: PLoS One. 2022 Jul 21;17(7):e0270736. doi: 10.1371/journal.pone.0270736 (PMC9302759; doi:10.1371/journal.pone.0270736)
Supplement: Attachment — Submitted filename: Response to reviewers.docx [file pone.0270736.s003.docx]

Dear dr. Neyrolles,

It is a great pleasure to submit to PLOS ONE our revised manuscript entitled ‘The metabolic hormone adiponectin affects the correlation between nutritional status and pneumococcal vaccine response in vulnerable indigenous children’.

We again thank the Editor and Reviewer for their constructive comments and suggestions. All extra suggestions made by the Reviewer as well as the editorial comments have been implemented in the revised manuscript. The explanatory details are listed below.

*Editor comments*

1. Please review your reference list to ensure that it is complete and correct. If you have cited papers that have been retracted, please include the rationale for doing so in the manuscript text, or remove these references and replace them with relevant current references. Any changes to the reference list should be mentioned in the rebuttal letter that accompanies your revised manuscript. If you need to cite a retracted article, indicate the article’s retracted status in the References list and also include a citation and full reference for the retraction notice.

We have meticulously gone through the reference list but could not find any retracted paper.

We have made one small adjustment to the reference list; in the methods section, we previously referred to the article of Verhagen et al. considering the determination of antibodies using fluorescent bead-based multiplex immunoassay, however, to fully capture the technical details of the assay, we now refer to the article of Stoof et al.

Following a previous comment of the editor, we have adjusted the title of our manuscript slightly to remove any implication of causality, into 'The metabolic hormone adiponectin affects the correlation between nutritional status and pneumococcal vaccine response in vulnerable indigenous children'.

*Reviewers’ comments*

1. Are there any data on invasive pneumococcal disease in the population studied or other indigenous populations? This would add to the justification for the study & would be useful to know

Although there are no specific data on invasive pneumococcal disease in our study population, high prevalence rates of acute respiratory tract infections and pneumococcal carriage rates have been observed in this population. These high rates are also seen in other indigenous populations. We have added this as extra information in the introduction section of our revised manuscript.

2. Minor editorial advices:

We have changed the grammatical adjustments accordingly. Also, we have moved lines 193-7 to the result section. Regarding the question why HMW adiponectin is measured rather than total levels; the Lumipulse assay used for HMW measurements is a very reliable method and it has previously been shown in a comparative analysis that total and HMW adiponectin have similar utility when assessing adiponectin levels in blood^1^.

We are confident that the current manuscript meets the quality criteria of PLOS ONE, and that the subject will be appreciated by the Journal’s readership.

Sincerely,

On behalf of all authors,

Lilly M. Verhagen
